# Engaging adults with severe mental illness in research: considerations and practical recommendations for meaningful patient and public involvement

**DOI:** 10.1186/s40900-026-00839-y

**Published:** 2026-02-26

**Authors:** Emily Shoesmith, Petal Petersen Williams, Jennifer Sweetman, Lisa Huddlestone, Jodi Pervin, Simon Hough, Susan Croft, Steven Dexter, Coleen Scothorne, Elena Ratschen

**Affiliations:** 1 Department of Health Sciences, University of York, York, UK; 2 Mental Health, Alcohol, Substance Use and Tobacco Research Unit, South African Medical Research Council, Cape Town, South Africa; 3 Institute for Life Course Health Research, Department of Global Health, Stellenbosch University, Stellenbosch, South Africa; 4 Patient and Public Involvement Panel Member, England, UK

**Keywords:** Patient and public involvement, Mental health, Severe mental illness, Smoking cessation, Inpatient care

## Abstract

**Background:**

Patient and public involvement (PPI) in mental health research is essential but presents unique challenges, particularly when engaging adults with severe mental illness (SMI). This report focusses on the process, benefits, and challenges of involving adults with lived experience of SMI as active partners within a five-year research programme aimed at co-producing a new evidence- and theory-based intervention to support smoking behaviour change following mental health inpatient care. It summarises our PPI strategy, the lived experience insight provided, and practical recommendations for facilitating meaningful involvement of people with SMI throughout the research process.

**Methods:**

We reflect on our approach to PPI within the research programme. Involvement spanned the entire research programme, from intervention design to dissemination. The research team reflected on the PPI participants’ experience via informal feedback, meeting notes, and reflective team discussions. These reflections informed the identification of practical considerations and recommendations.

**Results:**

PPI was crucial to the research and had a powerful, meaningful impact, with group members playing a key role in shaping participant-facing materials and intervention resources. Success was supported by early planning, dedicated facilitation, sustained relationship-building, and clear communication. Challenges included balancing meaningful input with accessibility, accommodating individual needs and preferences, and supporting varying levels of engagement due to participants’ ongoing mental health experience.

**Conclusion:**

Involving adults with SMI in research is both feasible and valuable, and requires careful planning, flexibility, and support. This report highlights the importance of relational approaches and tailored support to enable meaningful contributions, offering practical guidance for researchers developing inclusive PPI strategies in mental health trials involving complex populations.

## Abstract (plain language summary)

**Background:**

People with severe mental illness (SMI) often face particular challenges, especially when it comes to being involved in research. However, their input is very important, especially for research that aims to support their health and wellbeing. This report describes how we involved people with lived experience of SMI in a five-year research programme. The goal of the research was to support people to stop smoking after leaving a mental health hospital.

**Methods:**

We involved people with lived experience of SMI (including one carer), at every stage of the research, from designing the intervention to sharing the results. The research team looked back on their experience, reviewed meeting notes, and had group discussions to reflect on what went well and what could be improved. These reflections helped the team to come up with recommendations for involving people with lived experience in future research.

**Results:**

It was important to build strong relationships, communicate clearly, and provide support throughout. It was important to ensure activities were accessible and flexible, especially as people’s ability to participate could change depending on their mental health. It was also important to adapt research activities to individual preferences and needs.

**Conclusion:**

Involving people with lived experience of SMI in research was both possible and valuable. Their voices helped make the research more relevant. Our experience shows that with the right support, people with lived experience can make important contributions to complex mental health studies. This short report offers recommendations to help other researchers plan meaningful and inclusive involvement in similar projects.

## Background

Patient and Public Involvement (PPI) among adults with severe mental illness (SMI), including those with bipolar and psychosis spectrum conditions, has gained increasing attention in research and policy [1, 2]. This reflects a broader shift toward recognising the value of involving individuals with lived experience in shaping research, co-producing knowledge, and informing the development of interventions intended for their population group. Increasingly, adults with lived experience of SMI are being invited to contribute to research processes as active partners. Their involvement has been shown to improve the relevance and acceptability of research methods and outputs but also the design, implementation, and uptake of new initiatives and interventions [3–7]. PPI promotes greater recognition of participatory approaches within the research community [8, 9], and can contribute to meaningful outcomes for PPI members, including increased empowerment, respect, and a sense of being valued [10, 11].

While reviews of PPI in mental health research have advanced understanding by defining core concepts [12, 13], outlining implementation strategies [14, 15], and identifying barriers, facilitators, and outcomes [15, 16], there remains a gap in our understanding of how PPI is implemented and experienced within specific research contexts. Recent literature has advanced the field by clarifying core concepts of co-design, stating that while collaborative, inclusive principles are increasingly recognised, there is still inconsistency in how frameworks and processes are applied and reported in practice [17, 18]. Participant involvement has broadened, with roles ranging from informants to active co-designers [9], and there’s a call for clearer, more transparent reporting to enable best practice and cumulative learning [19]. The role of PPI in smoking cessation research involving people with lived experience of SMI remains underexplored. While there is a need for updated data on smoking prevalence among adults with SMI in the United Kingdom, recent data from a large-scale international and multi-country survey in South Asia confirm that tobacco use rates in this population remain substantially elevated compared to the general population [20]. A recent meta-analysis of 19 pooled studies found global tobacco/nicotine use disorder prevalence rates in this population between 33 and 65% [21]. Given the complex needs of this population and the currently known high prevalence of tobacco dependence [22], effective and context-specific implementation of PPI, particularly in smoking cessation studies with people with lived experience of SMI, remains underexplored. Documenting and sharing lessons learned from PPI initiatives in this area is essential for: informing best practices, facilitating replication, and ensuring that future research is both relevant to, and appropriate for, those it seeks to serve, across both mental health inpatient and community settings [23].

Tobacco dependence among individuals with lived experience of SMI is shaped by intersecting social, psychological, and environmental factors [24, 25] including unstable housing, limited access to supportive networks, stigma, and high-stress living conditions, which can impact sustained engagement and complicate how lived experience is integrated into research design and decision-making. Specific challenges include uncertainty around how to best incorporate insights from those with personal experience of tobacco dependence and mental health care, especially when research agendas are shaped by clinical or regulatory priorities [26]. Involvement may also be constrained by different perspectives on tobacco dependence, especially in situations where smoking may sometimes still be perceived as an important coping mechanism [27, 28] or perceived as one of the few areas in which they exercise personal agency within environments where autonomy can be limited. Practical challenges are also evident, as factors such as fluctuating mental health, fatigue, and unplanned hospital admissions may complicate sustained involvement throughout projects [16].

In light of these challenges, this report draws on lessons learned from establishing and sustaining a Patient and Public Involvement (PPI) panel of adults with lived experience of severe mental illness (SMI) over the five-year *SCEPTRE* research programme—*Promoting Smoking Cessation and Preventing Relapse to Tobacco Use Following a Smokefree Mental Health Inpatient Stay* [29, 30]. The sustained five-year commitment was critical to ensuring meaningful engagement and continuity as the project evolved from co-design to feasibility testing. This report outlines practical insights and recommendations for developing, maintaining, and embedding long-term PPI partnerships within complex mental health research, to inform future SMI-specific smoking cessation and relapse prevention initiatives.

### Overview of the SCEPTRE programme

Many healthcare settings now implement smokefree policies and offer smoking cessation support during inpatient admissions [31]. However, relapse rates post-discharge remain high, with up to 76% of individuals resuming smoking within one day [32], largely due to a lack of continued support [29]. This limits the long-term impact of inpatient support and highlights a critical research gap: the need for effective strategies to sustain positive smoking behaviour change at the point of discharge [33].

The SCEPTRE programme was designed to address this gap by applying established behaviour change frameworks [34–36] to develop a theory- and evidence-informed complex intervention aimed at supporting individuals with a mental health condition in maintaining smoking behaviour change post-discharge from inpatient care [29]. A central component of the programme was the establishment of a dedicated PPI panel, ensuring the perspectives of people with lived experience of SMI were integrated throughout the research process.

## Methods

Ethics approval for the SCEPTRE PPI activities and all research involving direct PPI input was granted by the North West - Greater Manchester West Research Ethics Committee (REC) (23/NW/0312).

### Establishment and formation of the SCEPTRE PPI panel

Recruitment was conducted through advertisements circulated in 2020 across participating NHS mental health Trusts involved in the wider study. Two PPI contributors were active co-applicants during grant development, contributing equally to the initial conception, design and justification of the SCEPTRE programme. These co-applicants continued to play a central role post-award, alongside subsequently recruited PPI panel members, in the co-production of intervention materials, participant resources, decision-making, and dissemination. This structure facilitated involvement in all major phases of the research, while also acknowledging the practical need for staged panel expansion. Eligibility criteria for joining the SCEPTRE PPI panel were: being age 18 or older, having lived experience of both tobacco dependence and a mental health inpatient stay (for service users), or being a carer of someone with SMI and tobacco dependence, and capacity to contribute to discussions. The panel comprised nine members: eight individuals with lived experience of tobacco dependence and a mental health inpatient stay, and one relative and carer of a person with SMI, with experience of tobacco dependence. Carer participation is important because carers typically have in-depth understanding of how SMI and tobacco dependence affects both the individual and their social network; yet carers’ perspectives on smoking in SMI remains under-represented in literature [37]. The term ‘members with lived experience’ hereafter denotes both service users and the carer participant. The term ‘serious mental illness’ (SMI) is used in this report to refer to mental health diagnoses that have substantial, lasting impact on individuals’ functioning and daily life. While there is ongoing debate about optimal language, ‘SMI’ was selected because it matched both research convention and the terminology most PPI members recognised from health services. The group did not express any negative feelings towards the term even when they supported the development of bias-free-language as part of our study.

The group was diverse in terms of gender (six women and three men), geographical location within England (Leeds, York, Chester, and Sheffield), and age (32–73 years). However, all panel members identified as White and information on socioeconomic status was not systematically collected. We acknowledge that greater ethnic and socioeconomic diversity would have further strengthened the panel’s representativeness. Ensuring broader demographic representation and systematic data collection are important priorities for future PPI work, especially given demographic patterns of SMI and smoking.

### Engagement of the SCEPTRE PPI panel

PPI was embedded across all phases and work packages of the SCEPTRE programme. PPI panel meetings were typically attended by one (occasionally two) academic researchers and the remainder of participants were individuals with lived experience of SMI or carers, ensuring that discussions and decisions were service user-led rather than balanced equally by researcher presence. Members of the SCEPTRE PPI panel were actively engaged in multiple roles and at all stages of the research, from initial design through to dissemination. Of the nine original members recruited, four (44%) remained actively involved throughout the programme’s five-year duration and are co-authors on this publication. Importantly, all final active members were from the original group of recruits. Given the complex health context and five-year timescale, this level of sustained engagement reflects both the challenges and the value of long-term relationship-building in PPI with adults experiencing severe mental illness. Engagement involved the co-production of the SCEPTRE intervention (described in detail elsewhere) [29], the development of participant-facing materials including information sheets and consent forms, and resources specifically designed to support the uptake and usability of the Smoke Free app [38]. This was achieved by incorporating lived-experience feedback into app instructions, ensuring accessible language and step-by-step guides, offering troubleshooting support during and after discharge, and tailoring motivational content to users’ real-world needs and preferences. Panel members also played a central role in the design and content development of the research programme’s public-facing website, helping to ensure the information presented was both accessible and reflective of service user priorities. In addition, the panel contributed to the creation of guidance promoting the use of inclusive language when introducing SCEPTRE to potential participants. Collectively, these activities reflect a sustained and meaningful model of involvement that prioritised co-creation and embedded lived experience at the core of the research. In line with recent definitions [17, 18], we understand co-creation as an overarching process in which people with lived experience take on equal roles alongside researchers at every stage of research, from priority-setting and co-design through to delivery and evaluation. In this framing, co-design refers specifically to collaborative activities focused on developing solutions or interventions, while co-production relates to the joint implementation and collaborative production of project outputs. Our model reflects a commitment to true co-creation, where the expertise of PPI contributors is embedded and valued throughout the entire research process.

### Process for gathering and synthesising reflections

Reflections reported in this report were collected using a multi-source, pragmatic approach throughout the research programme. Data sources included (a) informal verbal and written feedback from PPI panel members (typically captured during or after meetings); (b) detailed notes recorded by the research team during PPI meetings and interactions; and (c) structured reflective discussions among the research team at regular intervals. These sources were reviewed and synthesised collaboratively by the research team with PPI member input to identify recurring themes, challenges, and practical recommendations for inclusive PPI, ensuring PPI contributors’ voices shaped the process itself. For instance, during the pilot, PPI members highlighted that some phrases in the participant information sheet felt inconsistent or emotionally loaded, such as repeated use of “wish”, the term “quit”, and wording that referred to participants being “completely happy” or “upset”. In response, the research team revised the documents to use consistent, less burdening language (for example, replacing “quit” with “changing smoking behaviour”, removing repeated “wish”, and reframing “passing on your views” as “sharing your views”) and clarified jargon such as “mixed-methods research” and programme acronyms, directly aligning materials with PPI preferences. One PPI member noted, “being part of a group where your contribution is being acknowledged is a good thing….although in many ways your life might be difficult, at least it can be useful”.

## Results

### Lessons learned and recommendations

During the development and planning of SCEPTRE, key considerations and practical strategies were identified as important for establishing and sustaining long-term engagement of the PPI panel. Practical recommendations for research teams and PPI members are summarised in Tables 1 and 2, respectively. A central lesson from SCEPTRE was that the quality of relationships between researchers and PPI members is central to meaningful and sustained engagement. Rather than viewing involvement as a series of tasks, the team adopted an approach grounded in trust, empathy, and flexibility. This was achieved by offering multiple participation modes (in-person, telephone, and email), allowing meeting times and formats to be adjusted based on members’ preferences and health needs, enabling panel members to participate as much or as little as they felt able at any given time, and regularly seeking feedback to adapt processes. This model not only supported the practicalities of collaboration but also created an environment where PPI members felt safe, valued, and empowered to contribute.

### Involvement from the start

Early engagement of people with lived experience of SMI is essential for fostering a sense of ownership and enabling equitable collaboration between researchers and PPI members. For example, PPI members were involved from the outset in the co-production of the SCEPTRE intervention, reinforcing their role as active partners rather than passive participants. Such early involvement appeared to support trust-building, clarified roles and expectations, and promoted sustained involvement. This was particularly important given the potential for fluctuating mental health and service engagement among individuals with lived experience of SMI.

### Roles and responsibilities

Establishing clear roles and responsibilities was essential to building an effective, respectful partnership with PPI members. Meaningful involvement relied on recognising and valuing the lived experience of contributors as a distinct form of expertise. As one PPI member stated to the PPI co-ordinator, “you have been very very good at encouraging us and praising us and making us feel valued”. Actively listening to their input, acting on their recommendations, and offering autonomy within defined tasks encouraged trust. A key lesson was that clarity from the outset improved both confidence and participation. Well-defined tasks helped PPI members to understand their role in shaping intervention content and supporting materials.

### Planning for relational continuity

Effective involvement of PPI members required a relationship-first model that went beyond brief, task-based transactional engagement. Sustaining the partnership required more than formal meetings. The team prioritised relational continuity, assigning consistent points of contact, especially with the PPI coordinator, and maintaining open channels of communication. Maintaining contact during periods of reduced activity was especially important. Regular check-in emails or telephone calls, typically every few weeks to monthly, but adjusted as needed according to individual circumstances, were used not only to share study updates, but also to sustain rapport and demonstrate ongoing commitment to the involvement process. PPI members reported that these points of contact helped to mitigate feelings of disconnection and reinforced the value placed on their contributions.

Given the emotional complexities associated with discussing smoking relapse and recovery in the context of SMI, PPI members occasionally disclosed relapse experience during check-ins, sometimes accompanied by feelings of shame or self-blame. These disclosures occurred within a PPI process where traditional power imbalances between researchers and lived-experience contributors may be heightened, for example, due to the academic authority held by the research team or the vulnerability associated with sharing setbacks. It was important to respond to such disclosures with empathy and non-judgement, openly acknowledging that relapse is a common and informative part of smoking cessation. The team also aimed to normalise discussions about relapse, not only to foster individual empowerment but also to disrupt the hierarchical dynamic that can discourage honest disclosure. By intentionally adopting a relational, rather than transactional approach and engaging in ongoing reflexivity about our own positionality and power, we aimed to create a supportive environment where PPI members felt safe to share both their setbacks and successes, ensuring all contributions, especially those that challenge conventional narratives, were viewed as valuable for intervention refinement. Recognising and discussing power, and creating opportunities for reciprocal learning, were central to enabling genuine, empowering involvement.

### Emphasise dual-expertise co-production

Members were recruited to the SCEPTRE PPI panel based on their lived experience of both mental health inpatient care and tobacco dependence, either through personal experience or in their role as a carer and relative. Their dual expertise was explicitly acknowledged and integrated throughout the study. Involvement was structured to validate and draw on this insight, particularly regarding how mental health symptoms may influence smoking behaviour. For example, SCEPTRE included the provision of a resource folder, co-produced with PPI members, containing practical and motivational content [29], such as success stories, activity planners, and techniques/activities that members had personally found effective when quitting smoking. These resources were developed for use by trial participants, with the aim of supporting and motivating those attempting to stop smoking as part of the intervention.

### Support, flexibility and inclusive engagement

A key challenge to sustained and meaningful involvement of PPI members with lived experience of SMI was the fluctuating nature of their mental health. Participants routinely informed the researchers when they could not participate, citing barriers such as low energy, emotional distress, and limited availability due to ongoing mental health support. These factors sometimes affected their capacity for consistent engagement. Reflecting on the five-year duration of the programme, it is important to acknowledge that such a long-term commitment inevitably encompassed a range of experiences. Our approach recognised this variability as a natural and expected part of living with SMI, and processes were designed to flexibly accommodate changing circumstances over time. PPI co-authors reflected that having flexibility in participation was key to maintaining their involvement and sense of ownership. To support wellbeing, safeguarding processes were in place throughout the programme. These included regular check-ins with panel members, such as one-to-one phone calls or emails to ask about their wellbeing and ongoing needs; flexible participation options, for example, allowing members to join meetings by phone or video, contribute to documents between meetings, or take breaks from involvement when unwell without penalty or pressure; flexible options for communication, for example post, email, telephone; accessible formats, for instance large print, plain language; and, when initial plans proved challenging, such as group feedback sessions that some found overwhelming, alternative options like written feedback or individual meetings were introduced; and clear pathways for raising concerns, such as providing direct contact details for the programme lead and PPI co-ordinator, and reminders that any concerns could be shared confidentially and addressed promptly.

It was also important to account for cognitive variability and motivational challenges within this group. To address this, tasks could be broken down into smaller components, regular check-ins were offered, and visual aids or infographics were often used to communicate research concepts. These approaches, some of which were suggested by PPI members themselves, also informed the co-design of participant-facing materials, such as information sheets and consent forms, and contributed to a more accessible and supportive research environment. Additionally, appropriately recognising PPI members’ time and expertise was central to our approach, and members were compensated for their contributions (£25 per hour). To respect individual circumstances, contributors could choose their preferred method of payment, either bank transfer (which could take 1–2 months to arrive due to university finance processes) or rapid delivery of high-street vouchers. Two core PPI members, named as co-applicants, were paid directly from the sponsor’s budget in alignment with their formal role. Payment was always provided after participation, with no expectation of future involvement, and was set at rates reflecting fair reward for expertise.

### Tackling health inequity as a shared PPI goal

Positioning smoking cessation as a health equity issue was a powerful motivator for SCEPTRE PPI members. Co-production was not only a methodological choice but a means of aligning research activities with shared values around health equity. For PPI members with SMI, who face disproportionately higher rates of tobacco dependence and related illness, engaging in this type of research held deeper meaning when explicitly connected to the goal of tackling these entrenched disparities. For example, during PPI meetings several members reflected on how historic neglect and under-resourcing of mental health and smoking cessation services for people with SMI contributed to preventable harm in their communities, seeing their contribution to SCEPTRE as an opportunity to make a difference for people like them, who are often overlooked in healthcare. The group co-developed resources that foregrounded this equity mission, such as success stories from people with SMI and tailored guidance on accessing stop smoking support in the context of mental health. More broadly, health equity in this context refers to intentionally designing, delivering, and evaluating services to address the higher burden of tobacco harm among people with SMI, an approach supported by national audits and reviews showing targeted services can help reduce health inequalities for vulnerable groups.

PPI members consistently expressed a desire for their lived experience to inform not just the intervention itself, but the wider systems in which care is delivered. Collaborative discussions frequently extended beyond specific research tasks to include critical reflections on how mental health services approach tobacco dependency. In particular, PPI members were motivated by the prospect that their involvement could help shift services away from permissive or ambivalent smoking cultures toward more proactive and supportive approaches to cessation.

Framing smoking cessation as a matter of health equity allowed PPI members to see their contributions as part of a collective effort to improve the quality of care for others with similar experiences. This shared goal appeared to help build meaning, especially when participants saw that their insights were valued not only for intervention refinement but also for shaping the values and direction of the research itself.

**Table 1:** Practical recommendations for research teams

Involve early and frequently	Work with PPI members at early stages where possible, including idea generation, co-developing research questions, intervention design and recruitment materials from the outset. It may not be possible in projects with a fixed protocol (e.g., secondary analysis of pre-collected data), regulatory constraints that preclude protocol change, or practical barriers such as short funding timelines.
Be clear and inclusive	Ensure shared understanding through accessible language and collaboratively clarified roles, expectations, and timelines from the outset.
Prioritise flexibility	Adapt involvement activities to account for fluctuating health, including offering remote participation and flexible meeting times.
Build trust	Invest time in relationship-building before expecting substantial input. Maintain relationships even when PPI involvement fluctuates due to health or other life events.
Recognise contributions	Acknowledge input through co-authorship, payment, or other formal recognition.
Define roles early	Clarify roles together early on and outline tasks, expectations, and boundaries from the beginning.
Keep tasks concrete and purposeful	Agree on concrete, purposeful activities together and avoid vague requests and ensure PPI members understand how their input will shape outcomes.
Act on feedback	Show how joint feedback and discussions informed project decisions and how suggestions are implemented to demonstrate that lived experience is valuable.
Dual-expertise integration	Recognise PPI members’ unique experience with both smoking and SMI (in this context) and actively seek their views on how the two interact.
Offer multiple communication formats	Provide information via email, post, phone, or in-person, based on individual preferences. Check preferences at the outset on how PPI members would like to receive materials and participate in meetings.
Use accessible materials	Ensure all written materials are available in large print, plain language, and alternative formats where needed.
Build flexibility into timelines	Anticipate fluctuations in involvement by allowing for pauses and re-engagement.
Establish a clear and supportive point of contact	Ensure a consistent point of contact such as a dedicated PPI coordinator to support collaboration and offer regular check-ins and support.
Avoid rigid adherence to initial plans	Be willing to revise project activities based on evolving needs or participant feedback.
Normalise and validate changing needs and support transition if required	Recognise that fluctuating mental health is expected, accommodate this in a non-judgemental and supportive environment and offer re-engagement options or alternative roles if someone steps away temporarily.
Recognise contributions through co-authorship and payment	Acknowledge shared contributions through co-authorship, recognition, or other equitable means to support motivation and ongoing engagement.

**Table 2:** Practical recommendations for PPI members

Ask questions early	Seek clarification on your role, expectations, and how your input will be used.
Share preferences and needs	Communicate preferred formats, availability, and support needs with the team.
Engage in co-learning and shared reflection with the team	View involvement as a two-way process where lived experience and research expertise are equally valued.
Use your voice – Don’t hesitate to raise concerns	Contribute ideas and reflections openly to strengthen the team’s shared learning.
Recognise the unique expertise you bring to the partnership	You bring unique insights; your knowledge of your lived experience is essential to creating realistic and appropriate interventions.
Stay connected	Maintain communication with the PPI coordinator or lead to help navigate changes in health or engagement.
Express preferences	Let the research team know how you’d like to receive materials and participate.
Request adjustments	If materials are difficult to read or access, ask for alternatives that better suit your needs.
Participate in your own way	Choose the level and mode of engagement that feels manageable.
Communicate when things change	If your mental health or schedule changes, it’s acceptable to step back temporarily. Keeping the team informed helps them to support you.
Rejoin when ready	If you pause your involvement, know that returning later is welcomed and supported.
Reflect on what helped	Share reflections on what helped the team work effectively together and what made your involvement easier or more meaningful.

## Discussion

This report outlines the considerations and practical recommendations associated with establishing and sustaining a PPI panel comprising individuals with lived experience of SMI in the context of smoking cessation research. The reflective analysis and recommendations in this report were developed collaboratively by the core research team, which comprised the project lead, PPI coordinator, programme managers, research assistant, and, where appropriate, clinical collaborators as well as PPI members. Team reflections were informed by meeting notes, informal PPI feedback, and periodic review of emerging issues. The SCEPTRE programme demonstrated the value of early, flexible, and sustained involvement of people with lived experience in co-producing a complex intervention designed to support smoking behaviour change following mental health inpatient discharge. Our learning, as researchers and PPI members, emphasises the importance of accessibility, trust-building, and support within PPI frameworks, but also the need for flexibility and adaptability necessary for populations facing intersecting challenges of mental health conditions and smoking. For PPI members, sustained involvement fostered confidence and empowerment, while for researchers it reinforced the importance of flexibility and balancing roles. Our reflections highlight the need to adopt a more tailored, relational and person-centred approach, particularly for individuals whose engagement capacity may fluctuate due to health status.

The considerations and practical recommendations presented here move beyond generic best practice by providing mental health- and smoking-specific adaptations to PPI engagement. This includes recommendations around relapse normalisation, flexibility and adaptability required for fluctuating mental health status, varying needs and preferences, and long-term relationship continuity. These shifts are not only practical adjustments but reflect a deeper commitment to valuing lived experience as central to intervention co-design.

While our findings are based on a single research programme, they offer transferable recommendations for similar studies involving population groups facing health inequities. Future work should continue to explore how context-specific co-production strategies can contribute to more equitable and effective health intervention development, particularly in domains such as smoking cessation, where mental health service users have historically been excluded or underserved [39]. One notable limitation of our PPI panel was the lack of ethnic diversity and the absence of systematic data collection on socioeconomic status. All members identified as White, and we did not collect formal socioeconomic data. Given the demographic patterns of SMI and smoking, ensuring broader representation and systematic demographic documentation should be prioritised in future PPI initiatives to enhance relevance and generalisability. Additionally, our panel composition may be subject to self-selection bias, as those most able to commit to long-term or recurrent involvement might not fully reflect the wider SMI population; engagement fluctuated due to health or personal reasons over the five-year period, potentially shaping which voices were sustained; and the intensity of PPI described may not be feasible in all settings, as it relied on dedicated resources, established support networks, and a particular organisational context. Finally, we included a carer as part of our lived-experience panel to ensure the project benefited from both service user and carer perspectives. Despite ongoing efforts and active collaboration with mental health trust networks, only one carer was recruited. We acknowledge this as a limitation, and it highlights the challenge of involving a wider range of carers in PPI panels, especially when recruitment relies on existing organisational networks. Despite this, the carer contributed enthusiastically and without challenge, and their participation was facilitated by a relational and inclusive group dynamic that actively valued their perspective alongside the service user members. A further methodological limitation is that our analysis drew on informal verbal and written feedback, meeting notes, and team reflections rather than systematically collected qualitative data (e.g. audio-recorded interviews or focus groups analysed using a formal analytic framework). This pragmatic approach, chosen to minimise burden on PPI members with fluctuating mental health, may have introduced selection and interpretation biases, as researchers necessarily decided what to document and how to synthesise it, and some perspectives may not have been fully captured or explored in depth. Although we sought to mitigate this by triangulating multiple data sources, inviting PPI members to review and refine emerging recommendations, and including long-standing PPI contributors as co-authors, the resulting account should be understood as a reflective narrative rather than a comprehensive, systematically analysed dataset. In addition, we did not use a validated framework for evaluating and reporting PPI, such as GRIPP2 or other structured PPI reporting tools. This limits the transparency, comparability, and reproducibility of our account, as our reflections are not mapped onto an established set of PPI process and outcome domains. Our choice of a more pragmatic, narrative approach was influenced by a desire to minimise burden on panel members and to prioritise relationship-centred work with people experiencing severe mental illness. However, there is a need for future research to more rigorously investigate and measure genuine co-creation from the perspectives of people with lived experience and carers. This could include the use of validated PPI evaluation questionnaires completed by PPI members themselves, in-depth qualitative interviews or focus groups exploring their perceived influence and partnership over time, and structured evaluation tools or reporting frameworks to assess both the quality of involvement and its impact on study processes and outcomes. Combining such approaches with relational, low-burden PPI models may help to capture the experiential dimensions of co-creation while also generating more comparable evidence about what meaningful involvement looks like in complex mental health research.

### Impact of PPI on the wider programme and contributors

The PPI panel had significant and wide-reaching impacts on the SCEPTRE research programme. Panel members made direct contributions to the design and content of the intervention, co-produced participant-facing materials (including information sheets, consent forms, and app resources), and played a central role in shaping the public-facing project website and guidance on the use of inclusive language. Feedback from the panel influenced the accessibility and relevance of study materials, recruitment approaches, and dissemination strategies, ensuring these better reflected service user priorities and lived experience. PPI feedback also led to concrete changes in the design of intervention resources. The panel’s insights led to the integration of additional support components for mental health fluctuation and relapse prevention, resulting in a more person-centred intervention. For example, panel members reported that the original savings tracker (“I’m saving for…”) and long-term goal-setting (e.g. five-year plans) could feel overwhelming during periods of poor mental health. The tracker was therefore restructured to focus on shorter-term, weekly savings over the support period, and goal-setting materials were revised to emphasise realistic, near-term goals. Similarly, “blank” journal pages were supplemented with prompts such as “What has been important to you?” and “What has been beneficial for you?”, following PPI members’ suggestion that explicit prompts would make reflection more manageable for people experiencing cognitive difficulties. For PPI contributors, involvement fostered a sense of empowerment and value, as reflected in informal feedback and sustained engagement. Members reported that sharing their experiences and seeing their input translated into real change was meaningful and motivating. For the research team, working closely with PPI contributors enhanced understanding of service user needs, improved communication skills, and highlighted the value of relational, flexible approaches to co-production. Overall, PPI was not only instrumental in the intervention’s development but also promoted shared ownership of the research process, strengthening the quality and implementation potential of the programme. These wider impacts are consistent with reported benefits of meaningful patient and public involvement in complex health research.

## Conclusion

Involving individuals with lived experience of SMI in smoking cessation and mental health research is both feasible and essential for developing interventions that are relevant and acceptable. The SCEPTRE programme demonstrates that meaningful PPI engagement relies on trust, early engagement, sustained relationships, and flexible, person-centred approaches. These lessons offer valuable guidance for future research seeking to engage people with lived experience of SMI in complex health intervention development, embed authentic co-production in all stages of intervention development, to systematically address barriers to diverse involvement, including representation and support for carers, and to prioritise action that addresses health inequalities through robust PPI. Implementing these principles will be critical for designing equitable and effective health interventions for people with SMI in smoking cessation and related fields.

## Data Availability

No datasets were generated or analysed during the current study.

## Abbreviations

PPI: Patient and Public Involvement
SCEPTRE: Promoting Smoking CEssation and PrevenTing RElapse to tobacco dependence following a smokefree mental health inpatient stay
SMI: Severe mental illness
